# Dysregulated expression levels of *APH1B* in peripheral blood are associated with brain atrophy and amyloid-β deposition in Alzheimer’s disease

**DOI:** 10.1186/s13195-021-00919-z

**Published:** 2021-11-03

**Authors:** Young Ho Park, Jung-Min Pyun, Angela Hodges, Jae-Won Jang, Paula J. Bice, SangYun Kim, Andrew J. Saykin, Kwangsik Nho, for the AddNeuroMed consortium and the Alzheimer’s Disease Neuroimaging Initiative

**Affiliations:** 1 Department of Neurology, Seoul National University Bundang Hospital and Seoul National University College of Medicine, Seongnam, Republic of Korea; 2 Department of Neurology, Uijeongbu Eulji Medical Center, Eulji University, Uijeongbu, Republic of Korea; 3 Institute of Psychiatry, Psychology & Neuroscience, King’s College London, London, UK; 4 Department of Neurology, Kangwon National University Hospital, Chuncheon, Republic of Korea; 5 Department of Radiology and Imaging Sciences, and the Indiana Alzheimer Disease Center, Center for Neuroimaging, Indiana University School of Medicine, Indianapolis, IN USA; 6 Department of Medical and Molecular Genetics, Indiana University School of Medicine, Indianapolis, IN USA; 7 Center for Computational Biology and Bioinformatics, Indiana University School of Medicine, Indianapolis, IN USA

**Keywords:** Alzheimer’s disease, Transcriptome, Blood, Expression quantitative trait locus, Genome-wide association study, Expression, Imaging

## Abstract

**Background:**

The interaction between the brain and periphery might play a crucial role in the development of Alzheimer’s disease (AD).

**Methods:**

Using blood transcriptomic profile data from two independent AD cohorts, we performed expression quantitative trait locus (*cis*-eQTL) analysis of 29 significant genetic loci from a recent large-scale genome-wide association study to investigate the effects of the AD genetic variants on gene expression levels and identify their potential target genes. We then performed differential gene expression analysis of identified AD target genes and linear regression analysis to evaluate the association of differentially expressed genes with neuroimaging biomarkers.

**Results:**

A *cis*-eQTL analysis identified and replicated significant associations in seven genes (*APH1B*, *BIN1*, *FCER1G*, *GATS*, *MS4A6A*, *RABEP1*, *TRIM4*). *APH1B* expression levels in the blood increased in AD and were associated with entorhinal cortical thickness and global cortical amyloid-β deposition.

**Conclusion:**

An integrative analysis of genetics, blood-based transcriptomic profiles, and imaging biomarkers suggests that *APH1B* expression levels in the blood might play a role in the pathogenesis of AD.

**Supplementary Information:**

The online version contains supplementary material available at 10.1186/s13195-021-00919-z.

## Introduction

Alzheimer’s disease (AD) has a strong genetic component with high heritability (~ 70%) [1]. Over the last 10 years, more than 30 genes/loci have been identified as associated with AD by large-scale genome-wide association studies (GWASs) and sequencing data analysis [2]. However, most of the GWAS signals are located in noncoding regions of the genome, and their functional impact on AD is as yet poorly understood [3].

The interaction between the brain and periphery might play a crucial role in the development and pathogenesis of AD [4]. AD is classically regarded as a brain disorder. Despite the debate, an imbalance between production and clearance of amyloid-β (Aβ) in the brain is a very early, often initiating, factor in AD [5]. However, increasing experimental and epidemiological evidence has suggested that manifestations of AD extend beyond the brain [4]. For example, Aβ is produced not only in brain cells but also in peripheral organs and tissues including the liver, muscles, and various blood and endothelial cells [6]. Furthermore, the CNS and peripheral pools of Aβ can interact; some Aβ peptides in the CNS are cleared by phagocytosis or proteolytic degradation, whereas others are released into the blood [4]. Some Aβ peptides in the blood are phagocytosed by peripheral immune cells; some are degraded by Aβ-degrading enzymes, and some are transported by carriers to peripheral organs or tissues where they are degraded or excreted [4]. Considering there is a close interaction of Aβ metabolism between the brain and the periphery and trait-associated single nucleotide polymorphisms (SNPs) are likely to be expression quantitative trait loci (*cis*-eQTL) [7], the integrative analysis of genetics, blood-based transcriptomic profiles, and neuroimaging AD biomarkers could provide an opportunity for assessing the complex interplay between the brain and the periphery in the pathogenesis of AD [8].

In this study, we performed a *cis*-eQTL analysis of significant AD-associated SNPs from a recent AD GWAS meta-analysis [3] to investigate the effects of the AD SNPs on blood gene expression levels and identify their potential target genes using blood transcriptomic profile data from two independent AD cohorts. We then performed gene-set enrichment and differential gene expression analyses of identified target genes and evaluated associations of differentially expressed genes with neuroimaging biomarkers for AD and plasma protein levels.

## Methods

### Participants

Individuals used in the study were non-Hispanic Caucasian participants (AD, mild cognitive impairment (MCI), and cognitively normal older adults (CN)) from the Alzheimer’s Disease Neuroimaging Initiative (ADNI) and AddNeuroMed cohorts as discovery and replication samples, respectively. The ADNI was launched in 2003 as a public-private partnership, led by Principal Investigator Dr. Michael W. Weiner [9]. The primary goal of ADNI has been to test whether serial MRI, PET, other biological markers, and clinical and neuropsychological assessment can be combined to accurately capture the progression of MCI and early AD. The AddNeuroMed is a cross-European, public/private consortium developed for AD biomarker discovery [10]. The ADNI participants were recruited in North America, whereas the AddNeuroMed participants were recruited in Europe. In both cohorts, participants were categorized into three diagnostic groups (CN, MCI, AD). AD was diagnosed clinically according to the NINCDS/ADRDA criteria for probable AD in ADNI and AddNeuroMed [11]. MCI was diagnosed when there was objective memory impairment but without meeting the criteria for dementia [9, 10]. Written informed consent was obtained at the time of enrollment and included permission for analysis and data sharing. The protocol and informed consent forms were approved by the Institutional Review Board at each participating site.

### Genotyping and imputation

Genome-wide genotyping was performed using Illumina GWAS array platforms (Illumina Human610-Quad BeadChip, Illumina HumanOmni Express BeadChip, and Illumina HumanOmni 2.5 M BeadChip) [12, 13]. *APOE* genotyping was separately conducted [12]. Using PLINK 1.9 (www.cog-genomics.org/plink2/) [14], we then performed standard quality control (QC) procedures for samples and SNPs as described previously [15]: (1) for SNP, SNP call rate < 95%, Hardy-Weinberg *P*-value < 1 × 10^− 6^, and minor allele frequency (MAF) < 1%; (2) for sample, sex inconsistencies, and sample call rate < 95%. In order to prevent spurious associations due to population stratification, we used multidimensional scaling analysis to select only non-Hispanic participants of European ancestry that clustered with HapMap CEU (Utah residents with Northern and Western European ancestry from the CEPH collection) or TSI (Toscani in Italia) populations [16, 17]. After QC procedures (Supplementary Fig. 1), because these cohorts used different genotyping platforms, we imputed un-genotyped SNPs separately in each platform using MaCH with the Haplotype Reference Consortium data as a reference panel [18, 19].

### Blood-based RNA expression microarray profiling

The PAXgene Blood RNA Kit (Qiagen Inc., Valencia, CA, USA) was used to purify total RNA from the whole blood [12, 20]. The Affymetrix Human Genome U219 Array (Affymetrix, Santa Clara, CA, USA) and the Illumina Human HT-12 v3 Expression BeadChips (Illumina Inc., San Diego, CA, USA) were used in ADNI and AddNeuroMed, respectively, for expression profiling. All probe sets were mapped to the human genome reference sequence (hg19). Raw expression values were preprocessed with the robust multi-chip average normalization method in ADNI [21] and the robust spline normalization method in AddNeuroMed [22]. We investigated discrepancies between the reported sex and sex determined from sex-specific gene expression data, including *XIST* and *USP9Y*. We also checked whether SNP genotypes were matched with genotypes predicted from gene expression data [23]. After QC (Supplementary Fig. 2), the RNA expression profiles, which contained 21,150 probes in ADNI and 5141 probes in AddNeuroMed, were pre-adjusted with batch effects and RNA integrity number values using linear regression analysis.

### Identification of target genes of GWAS SNPs from eQTL analysis

As AD-associated SNPs, we used 29 independent SNPs from 29 distinct loci that showed genome-wide significant associations (*P* < 5 × 10^− 8^) in a recent AD GWAS meta-analysis [3]. Then, we selected genes that were located within ±1 M bp of 29 genome-wide significant SNPs and performed a *cis*-eQTL analysis using imputed GWAS and blood gene expression data to identify target genes as significantly associated with 29 genome-wide significant SNPs (false discovery rate (FDR)-corrected *P* < 0.05) [24] in ADNI and AddNeuroMed as discovery and replication samples, respectively.

### Pathway-based enrichment and differential gene expression analyses

We performed gene-set enrichment analysis to identify the biological pathways of AD-relevant target genes that were identified in ADNI and replicated in AddNeuroMed using three categories (biological process, molecular function, and cellular component) of the Gene Ontology (GO) resource [25, 26] and Enrichr (https://maayanlab.cloud/Enrichr/) [27]. We selected significantly enriched terms (FDR-corrected *P* < 0.05) that have at least 2 genes. We then performed an analysis of covariance (ANCOVA) and made violin plots to investigate which genes were differentially expressed between AD, MCI, and CN (FDR-corrected *P* < 0.05) and examined whether the ADNI findings were replicated in AddNeuroMed. We used age and sex as covariates.

### Assessment of the causal effect of differentially expressed genes on AD

We performed GWAS summary data-based Mendelian randomization (SMR) analysis [28] to assess the causal effect of differentially expressed genes on AD using eQTLGen data for peripheral blood [29] and a recent large-scale GWAS summary data for AD [3]. We also performed a HEIDI (heterogeneity in dependent instruments) test to distinguish pleiotropy from linkage [28].

### Association of differentially expressed genes with neuroimaging AD biomarkers and plasma-based protein levels

Hippocampal volume and entorhinal cortical thickness were measured as MRI biomarkers from T1-weighted brain MRI scans using FreeSurfer version 5.1 (surfer.nmr.mgh.harvard.edu) in both cohorts [30]. We performed a linear regression analysis to evaluate whether expression levels of differentially expressed genes were associated with hippocampal volume and entorhinal cortical thickness in ADNI (FDR-corrected *P* < 0.05) and to examine whether the ADNI findings were replicated in AddNeuroMed. We used age, sex, intracranial volumes, and MRI field strength as covariates. We also used a linear mixed model to jointly analyze the pooled individual imaging data from the ADNI and AddNeuroMed cohorts. Furthermore, global cortical amyloid deposition, as mean standardized uptake values, was measured as an amyloid PET biomarker using preprocessed (coregistered, averaged, standardized image and voxel size, uniform resolution) [^18^F] florbetapir PET scans with a whole cerebellum reference region in ADNI [31]. We performed linear regression analysis to evaluate whether expression levels of the differentially expressed genes were associated with brain amyloid deposition (FDR-corrected *P* < 0.05). We used age and sex as covariates. Amyloid biomarkers were not available in AddNeuroMed.

As plasma-based protein levels, 1001 proteins were measured using SOMAscan (SomaLogic, Inc., Boulder, CO, USA) Multiplexed Proteomic technology in the AddNeuroMed cohort [32]. We performed linear regression analysis to evaluate whether expression levels of differentially expressed genes were associated with plasma protein levels. We then performed ANCOVA to investigate which plasma proteins were differentially expressed between AD, MCI, and CN with age and sex as covariates.

### Association of differentially expressed genes with progression of MCI to AD dementia

We assessed the hazard ratio of expression levels of differentially expressed genes using Cox regression analysis with the follow-up time as a time variable and progression of MCI to AD dementia during follow-up period up to 5 years as a status variable. Covariates included age and sex.

In this study, we used R version 3.6.3 (R-project.org) for analysis unless otherwise specified. All statistical tests are two-sided unless otherwise specified.

## Results

A total of 1335 participants were included from two independent cohorts (661 from the ADNI and 674 from AddNeuroMed) in this study (Table 1). Using imputed GWAS and blood-based RNA gene expression data, we discovered that 29 genome-wide significant SNPs were eQTLs of 30 genes (*ADAMTS4*, *AIF1*, *APH1B*, *ARF4*, *AURKA*, *BIN1*, *CD55*, *CHRNE*, *CNN2*, *CSTF1*, *EPHA1*, *FAM63B*, *FCER1G*, *GATS*, *HLA-DRB1*, *MS4A4A*, *MS4A6A*, *MYBBP1A*, *NDUFS2*, *NUP88*, *PVRIG*, *RABEP1*, *SCIMP*, *SLC24A4*, *TAF6*, *TAP2*, *TRIM4*, *ZCWPW1*, *ZKSCAN1*, *ZNF668*; Supplementary Table 1) in ADNI and 15 genes (*APH1B*, *BIN1*, *CLPTM1*, *FCER1G*, *GATS*, *HLA-DQA1*, *HLA-DRA*, *HSPA6*, *ITGAX*, *MS4A6A*, *PILRB*, *RABEP1*, *RXRB*, *SNRPD2*, *TRIM4*; Supplementary Table 2) in AddNeuoMed. Seven genes (*APH1B*, *BIN1*, *FCER1G*, *GATS*, *MS4A6A*, *RABEP1*, *TRIM4*) were identified in ADNI and replicated in AddNeuroMed after adjusting for multiple testing.

**Table 1:** Demographics of study samples

Cohort	Diagnosis	*N*	Female (%)	Age at blood sample collection, mean (*SD*)	RIN, mean (*SD*)
ADNI (*N* = 661)	CN	213	107 (50%)	76.4 (6.4)	6.91 (0.51)
	MCI	345	144 (42%)	73.2 (7.9)	6.98 (0.55)
	AD	103	38 (37%)	77.6 (7.8)	6.98 (0.64)
AddNeuroMed (*N* = 674)	CN	243	147 (60%)	74.2 (6.6)	8.96 (0.73)
	MCI	208	120 (58%)	75.5 (6.5)	8.50 (0.59)
	AD	223	146 (65%)	76.8 (6.8)	8.43 (0.64)

The table was modified from a previous study [51]

*Abbreviations*: *AD* Alzheimer’s disease; *CN* cognitively normal older adults; *MCI* mild cognitive impairment; *RIN* RNA integrity number; *SD* standard deviation

Gene-set enrichment analysis of these seven genes revealed five significant pathways (protein oligomerization, positive regulation of programmed cell death, positive regulation of apoptotic process, vesicle-mediated transport, and regulation of apoptotic process) in the category of GO biological process and one significant pathway (protein homodimerization activity) in the category of GO molecular function (Table 2). No significant pathways were identified in the category of GO cellular component.

**Table 2:** Biological pathways identified in the enrichment analysis

Category	Pathway	Overlap	*P*-value	FDR-corrected *P*-value	Odds ratio	Gene
Biological process	Protein oligomerization	2/217	2.37 × 10^−3^	7.81 × 10^− 3^	26.33	*FCER1G*, *TRIM4*
	Positive regulation of programmed cell death	2/257	3.31 × 10^−3^	7.81 × 10^− 3^	22.23	*APH1B*, *BIN1*
	Positive regulation of the apoptotic process	2/307	4.69 × 10^−3^	7.81 × 10^− 3^	18.61	*APH1B*, *BIN1*
	Vesicle-mediated transport	2/410	8.22 × 10^−3^	1.03 × 10^−2^	13.94	*BIN1*, *RABEP*
	Regulation of the apoptotic process	2/815	3.04 × 10^−2^	3.04 × 10^− 2^	7.01	*APH1B*, *BIN1*
Molecular function	Protein homodimerization activity	2/664	2.07 × 10^−2^	2.07 × 10^− 2^	8.61	*FCER1G*, *RABEP1*

Overlap means a ratio of the number of input genes to the number of genes associated with each biological pathway. *P*-values and odds ratios were obtained from Fisher’s exact test

*Abbreviation*: *FDR* false discovery rate

**Fig. 1:**
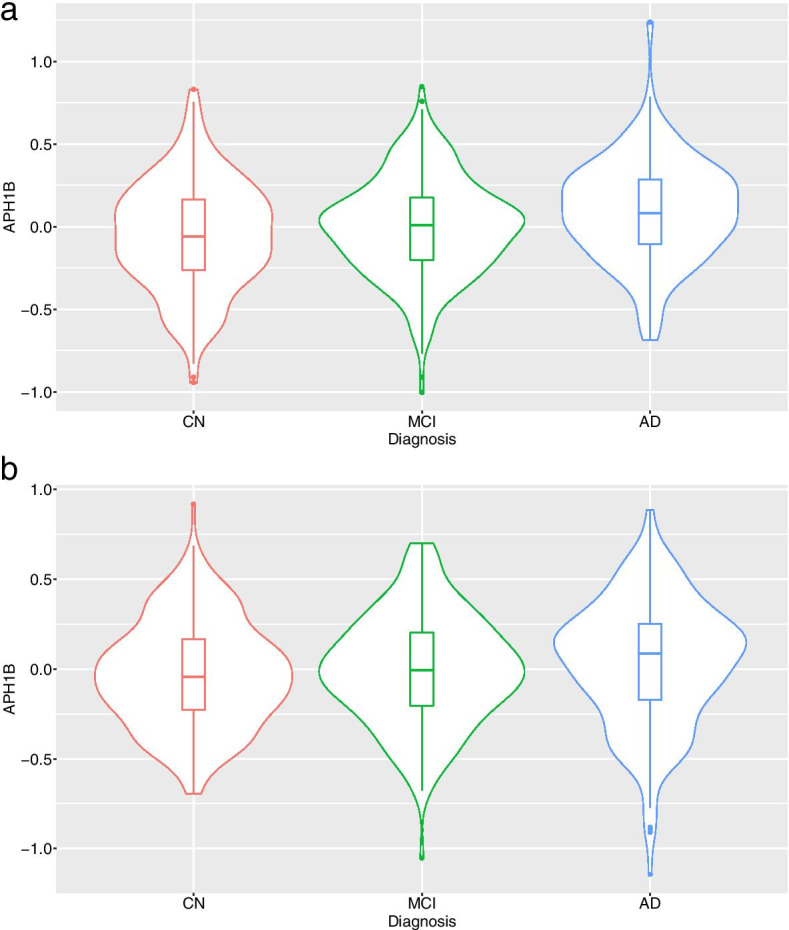
Violin plots for the blood expression levels of *APH1B* between CN, MCI, and AD. The violin plots show the probability density of the blood expression levels of *APH1B* as well as median and interquartile ranges in ADNI (**a**) and AddNeuroMed (**b**). The transcript identifier for *APH1B* was 11720068_a_at in ADNI and ILMN_1767816 in AddNeuroMed, respectively. Abbreviation: AD, Alzheimer disease; ADNI, Alzheimer’s Disease Neuroimaging Initiative; CN, cognitively normal older adults; MCI, mild cognitive impairment

In ADNI, differential gene expression analysis showed that expression levels of *APH1B* significantly increased in AD compared to CN (Supplementary Table 3) after adjusting for multiple testing, and this finding was replicated in AddNeuroMed (Fig. 1). In SMR analysis, we found that *P*_SMR_ of *APH1B* was 1.21 × 10^− 7^, which passed the experiment-wise significance threshold (*P*_SMR_ < 3.18 × 10^− 6^) and suggested the causal effect of *APH1B* expression on AD. *P*_HEIDI_ of *APH1B* was 0.053, which also passed the HEIDI test (*P*_HEIDI_ ≥ 0.05).

**Fig. 2:**
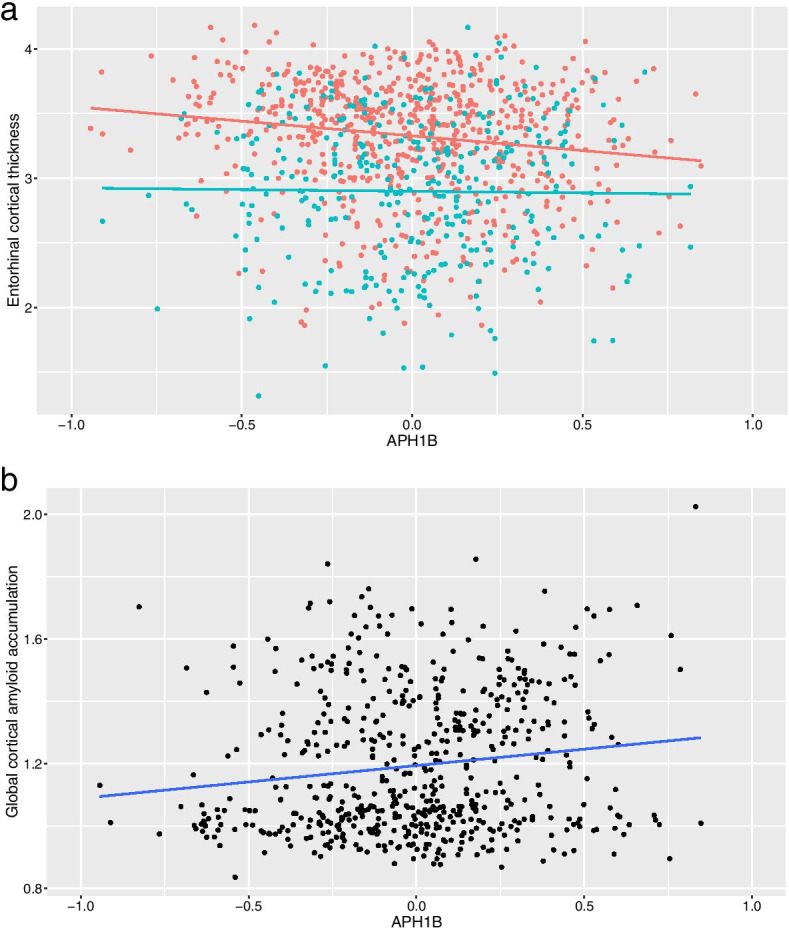
Relationship between the blood expression levels of *APH1B* and neuroimaging biomarkers. The association of the blood expression levels of *APH1B* with entorhinal cortical thickness (**a**) and averaged cortical uptake of [^18^F] florbetapir PET (**b**) was represented in the scatter plot. In panel **a**, the orange and light blue dots denote data from ADNI and AddNeuroMed, respectively. The orange and light blue lines were obtained from a linear regression analysis in ADNI and AddNeuroMed, respectively. In panel **b**, the black dots denote data from ADNI and the dark blue line was obtained from a linear regression analysis. The gray zones around the lines indicate a 95% confidence interval. The transcript identifier for *APH1B* was 11720068_a_at in ADNI and ILMN_1767816 in AddNeuroMed, respectively. Abbreviation: ADNI, Alzheimer’s Disease Neuroimaging Initiative

In addition, expression levels of *APH1B* were significantly associated with hippocampal volume and entorhinal cortical thickness in ADNI but not in AddNeuroMed (Table 3 and Fig. 2). However, when the pooled individuals from the ADNI and AddNeuroMed cohorts were jointly analyzed using a linear mixed model, expression levels of *APH1B* were associated with entorhinal cortical thickness but not with hippocampal volume. Furthermore, expression levels of *APH1B* were significantly associated with global cortical amyloid deposition in ADNI (Table 3 and Fig. 2).

**Table 3:** Association of the expression levels of *APH1B* with neuroimaging biomarkers in ADNI and AddNeuroMed

Cohort	Neuroimaging biomarker	Transcript ID	*T*-value	*P*-value	FDR-corrected *P*-value
ADNI	Entorhinal cortical thickness^a^	11720068_a_at	−2.85	4.50 × 10^−3^	2.25 × 10^−2^
		11740603_a_at	−2.36	1.87 × 10^−2^	4.67 × 10^− 2^
		11740602_s_at	−0.482	6.30 × 10^−1^	7.74 × 10^− 1^
		11720067_a_at	0.389	6.98 × 10^−1^	7.74 × 10^− 1^
		11740601_a_at	0.287	7.74 × 10^−1^	7.74 × 10^− 1^
	Hippocampal volume^a^	11720068_a_at	−2.69	7.40 × 10^−3^	3.70 × 10^− 2^
		11740603_a_at	−1.96	5.06 × 10^−2^	1.27 × 10^− 1^
		11740602_s_at	−0.104	9.17 × 10^−1^	9.69 × 10^− 1^
		11720067_a_at	−0.0969	9.23 × 10^−1^	9.69 × 10^− 1^
		11740601_a_at	0.0384	9.69 × 10^−1^	9.69 × 10^− 1^
	Averaged cortical uptake of [^18^F] florbetapir PET^b^	11720068_a_at	3.15	1.71 × 10^−3^	8.54 × 10^−3^
		11740603_a_at	2.64	8.51 × 10^−3^	2.13 × 10^−2^
		11740602_s_at	1.01	3.14 × 10^−1^	5.23 × 10^−1^
		11740601_a_at	0.765	4.45 × 10^−1^	5.56 × 10^− 1^
		11720067_a_at	0.121	9.04 × 10^−1^	9.04 × 10^− 1^
AddNeuroMed	Entorhinal cortical thickness^c^	ILMN_1767816	−0.761	4.47 × 10^−1^	N/A
	Hippocampal volume^c^	ILMN_1767816	0.454	6.50 × 10^−1^	N/A
ADNI and AddNeuroMed	Entorhinal cortical thickness^d^	11720068_a_at, ILMN_1767816	−2.96	3.18 × 10^−3^	N/A
	Hippocampal volume^d^		−1.93	5.91 × 10^−2^	N/A

^a^Data for 4 participants were unavailable (*n* = 657). *T*-value and *P*-values were obtained from linear regression analysis with adjustment of age, sex, intracranial volumes, and MRI field strength

^b^Data for 76 participants were unavailable (*n* = 585). *T*-value and *P*-values were obtained from linear regression analysis with adjustment of age and sex

^c^Data for 216 participants were unavailable (*n* = 458). *T*-value and *P*-values were obtained from linear regression analysis with adjustment of age, sex, and intracranial volumes

^d^Data for 220 participants were unavailable (*n* = 1115). *T*-value and *P*-values were obtained from a linear mixed model with adjustment of age, sex, and intracranial volumes

*Abbreviations*: *ADNI* Alzheimer’s Disease Neuroimaging Initiative; *FDR* false discovery rate; *Transcript ID* transcript identifier of Affymetrix Human Genome U219 Array for ADNI and Illumina Human HT-12 v3 Expression BeadChips for AddNeuroMed

With regard to plasma-based protein levels, 331 participants were available in AddNeuroMed. Expression levels of *APH1B* were associated with plasma levels of neuropilin-1, tropomyosin receptor kinase A, growth hormone receptor, and apolipoprotein A1 (Table 4). Particularly, levels of growth hormone receptor were significantly decreased in AD and MCI compared to CN (Table 4).

**Table 4:** Association of plasma biomarkers with the expression levels of *APH1B* and their different levels between AD, MCI, and CN in AddNeuroMed

Protein	*T*_*APH1B*_	*P*_*APH1B*_	*T*_*MCI*_	*P*_*MCI*_	*T*_*AD*_	*P*_*AD*_
Neuropilin-1	3.95	9.69 × 10^−5^	− 0.0657	9.48 × 10^− 1^	0.845	3.99 × 10^− 1^
Tropomyosin receptor kinase A	3.76	2.00 × 10^−4^	− 0.571	5.68 × 10^− 1^	1.54	1.24 × 10^− 1^
Growth hormone receptor	−3.59	3.80 × 10^− 4^	− 2.44	1.51 × 10^− 2^	− 3.25	1.29 × 10^− 3^
Apolipoprotein A1	− 3.53	4.82 × 10^− 4^	−0.448	6.54 × 10^− 1^	− 1.09	2.78 × 10^− 1^

Data for 344 participants were unavailable. *T*_*APH1B*_ and *P*_*APH1B*_ values were obtained from linear regression analysis for the expression levels of *APH1B* with adjustment of age and sex. Results with uncorrected *P* < 0.001 are presented. *T*_*MCI*_, *T*_*AD*_, *P*_*MCI*_, and *P*_*AD*_ were obtained from analysis of covariance for diagnosis group (CN, MCI, and AD) with adjustment of age and sex

*Abbreviations*: *AD* Alzheimer’s disease; *CN* cognitively normal older adults; *MCI* mild cognitive impairment; *P*_*APH1B*_ *P*-value for *APH1B* expression in linear regression analysis; *P*_*AD*_ *P*-value for AD diagnosis in the analysis of covariance; *P*_*MCI*_ *P*-value for MCI diagnosis in the analysis of covariance; *T*_*APH1B*_ *T*-value for *APH1B* expression in linear regression analysis; *T*_*AD*_ *T*-value for AD diagnosis in the analysis of covariance; *T*_*MCI*_ *T*-value for MCI diagnosis in the analysis of covariance

In Cox regression analysis, we used 320 and 186 patients with MCI in ADNI and AddNeuroMed, respectively, after excluding MCI without follow-up data. Among them, 61 and 47 MCI patients converted to AD in ADNI and AddNeuroMed, respectively, within a 5-year period after baseline. The hazard ratio (95% confidence interval) of expression levels of *APH1B* was 2.52 (1.02–6.25) in ADNI, whereas expression levels of *APH1B* were not significant in AddNeuroMed (*HR* = 0.765).

## Discussion

In this study, we identified and replicated seven target genes of 29 AD susceptibility SNPs of a recent large-scale GWAS by performing an eQTL analysis of blood transcriptomic profiles from two independent cohorts including AD, MCI, and CN. The biological pathways enriched in the seven genes included programmed cell death, protein oligomerization, and vesicle-mediated transport. Among the seven genes, expression levels of *APH1B* increased in the blood of AD patients and were associated with entorhinal cortical thickness; global cortical amyloid deposition; several plasma-based protein levels, including growth hormone receptors; and progression from MCI to AD dementia.

Programmed cell death or apoptosis, one of the biological pathways altered in peripheral blood from our study, has been commonly found in neurons and glial cells in AD [33]. Amyloid precursor protein intracellular domain (AICD), which is an amyloid precursor protein (APP)-derived cleavage product, was known to induce apoptosis in the pathogenesis of AD [34]. With respect to blood cells, the lymphocyte content of APP and apoptosis of lymphocytes are increased in patients with AD [35, 36]. The peripheral adaptive immune system including lymphocytes was reported to restrain AD pathology by clearance of Aβ [37]. Protein oligomerization plays a crucial role in the pathogenesis of AD [38]. In blood, the oligomerization tendency of Aβ is a diagnostic biomarker of AD [39] and is associated with neurodegenerative structural changes on MRI [40]. Vesicle-mediated transport includes various cellular transport processes using membrane-bounded vesicles. Previously, peripheral blood gene expression analysis showed that Fc-gamma receptor-mediated phagocytosis is dysregulated in AD [8].

*APH1B* gene encodes Aph-1b protein, one of the four subunits (Aph-1, nicastrin, presenilin, and Pen-2) of γ-secretase [41]. γ-secretase is present in many human tissues and well known for contributing to the pathogenesis of AD by cleaving APP and catalyzing the formation of Aβ [42]. Humans have two Aph-1 homologs, Aph-1a and Aph-1b [43], which differ in γ-secretase activity and production of longer and shorter Aβ peptides [44]. Compared to Aph-1a γ-secretase complexes, Aph-1b γ-secretase complexes produced more Aβ peptides with higher Aβ_1–42/1–40_ ratio in a mouse AD model [44]. Biochemical evaluation of pathogenic presenilin and APP mutations of familial AD suggested that relative increases in longer Aβ species are more relevant to AD than an absolute increment in total Aβ load [45]. It is known that human *APH1B* is more expressed in the blood than in the brain (https://www.proteinatlas.org) [46]. Our study showed that expression levels of *APH1B* increased in the blood of AD patients and eQTL of *APH1B* in the blood is one of 29 AD-associated SNPs from a recent large-scale AD GWAS meta-analysis [3]. SMR analysis also validated the causal effect of blood *APH1B* expression on AD. It suggests the possibility that Aβ peptides with higher Aβ_1–42/1–40_ ratio might be produced in the blood and transported into the brain in AD patients, which could be implicated in the pathogenesis of AD. Of note, *APH1B* was suggested as a causal gene of AD in a recent large-scale genome-wide meta-analysis [47].

Higher expression levels of *APH1B* were associated with lower plasma levels of growth hormone receptors, which decreased in patients with MCI and AD compared to CN. The effects of growth hormone are exerted by binding to the growth hormone receptors on target cells, which stimulate the production and secretion of insulin-like growth factor 1 (IGF-1) from the liver and other tissues [48]. The deficits in IGF-1 signaling have been related to AD pathology such as increased accumulation of Aβ, phosphorylated tau, increased neuroinflammation, and apoptosis [49]. Despite evidence for the involvement of the IGF-1 signaling pathway, the human growth hormone secretagogue failed to show efficacy for slowing the rate of progression of AD in a previous clinical trial [50]. Decreased plasma levels of growth hormone receptors in patients with MCI and AD from our study might be related to deficits in the IGF-1 signaling pathway in AD. In addition, the growth hormone receptor is known to be one of the γ-secretase substrates [51], which might explain the relationship between decreased plasma levels of growth hormone receptors and increased expression levels of *APH1B* in our study.

## Limitations

This study has some limitations. First, as eQTL analysis identified and replicated seven AD target genes, we could use only seven genes in the pathway enrichment analysis. Although there is no general rule regarding the number of genes, a list of tens or hundreds of genes is commonly used for enrichment analysis. Second, blood-based RNA expression profiles could be influenced by confounding factors such as medication, as well as blood collection, processing, and storage procedures. The transcriptomic samples in the ADNI and AddNeuroMed cohorts were, however, collected, processed, and stored following the standard protocols to minimize these risks [52]. Third, RNA expression profiling was performed on different microarray platforms in the ADNI and AddNeuroMed cohorts. Therefore, we did not perform a mega-analysis but used two cohorts as discovery and replication samples in this study. Fourth, as amyloid biomarkers were not available in AddNeuroMed, the association of expression levels of *APH1B* with brain amyloid deposition was investigated in only ADNI.

## Conclusions

In summary, our results show that dysregulated expression levels of *APH1B* in peripheral blood are associated with brain atrophy and Aβ deposition in AD. Considering the complex interaction between the brain and the periphery, *APH1B* expression levels in the blood might play a role in the pathogenesis of AD. Although γ-secretase has been a target for therapeutic development for AD, all of the γ-secretase inhibitors until now have failed to show efficacy in clinical trials [53]. Because γ-secretase cleaves more than 100 substrates besides APP [54], it would be extremely difficult to obtain a safe therapeutic window with blocking indiscriminately all different γ-secretase complexes [45]. Previously, selective targeting of Aph-1b γ-secretase complexes was suggested as an effective treatment option for AD [44]. Deficiency of Aph-1a was associated with reduced γ-secretase activity, whereas deficiency of Aph-1b was not, which indicates that Aph-1b γ-secretase complexes may fulfill redundant functions [43]. Replication analysis in independent larger cohorts and a functional study of *APH1B* and its implication for AD treatment are warranted.

## Supplementary Information

**Additional file 1: Supplementary TableS 1**. Blood eQTL data generated from GWAS genotyping and blood-based RNA expression data in ADNI. **Supplementary Table S2**. Blood eQTL data generated from GWAS genotyping and blood-based RNA expression data in AddNeuroMed. **Supplementary Table S3**. Results of differential gene expression analysis between AD, MCI and CN in ADNI

**Additional file 2: Supplementary Figure S1**. GWAS quality control procedures for samples in the Alzheimer’s Disease Neuroimaging Initiative cohort. **Supplementary Figure S2**. Quality control procedures of mRNA expression data for samples in the Alzheimer’s Disease Neuroimaging Initiative cohort

## Data Availability

The datasets used and/or analyzed during the current study are available from the corresponding author on reasonable request. Data used in the preparation of this article were obtained from the Alzheimer’s Disease Neuroimaging Initiative (ADNI) database (adni.loni.usc.edu). As such, the investigators within the ADNI contributed to the design and implementation of ADNI and/or provided data but did not participate in the analysis or writing of this report. A complete listing of ADNI investigators can be found at http://adni.loni.usc.edu/wp-content/uploads/how_to_apply/ADNI_Acknowledgement_List.pdf.
