# Supplementary material for: Dysregulated expression levels of APH1B in peripheral blood are associated with brain atrophy and amyloid-β deposition in Alzheimer’s disease
Source: Alzheimers Res Ther. 2021 Nov 3;13:183. doi: 10.1186/s13195-021-00919-z (PMC8567578; doi:10.1186/s13195-021-00919-z)
Supplement: Supplementary file 1 — Additional file 1: Supplementary TableS 1. Blood eQTL data generated from GWAS genotyping and blood-based RNA expression data in ADNI. Supplementary Table S2. Blood eQTL data generated from GWAS genotyping and blood-based RNA expression data in AddNeuroMed. Supplementary Table S3. Results of differential gene expression analysis between AD, MCI and CN in ADNI [file 13195_2021_919_MOESM1_ESM.pdf]

**Supplementary Table 1. Blood eQTL data generated from GWAS genotyping and blood-based RNA expression data in ADNI**

| Chr | SNP | A1 | A2 | MAF | $Z_{GWAS}$ | $P_{GWAS}$ | Transcript ID | Gene | $T_{eQTL}$ | $P_{eQTL}$ | FDR-corrected $P_{eQTL}$ |
|---|---|---|---|---|---|---|---|---|---|---|---|
| 1 | rs4575098 | A | G | 0.228026 | 6.35743578 | $2.05\times10^{-10}$ | 11760705_a_at | *NDUFS2* | -4.0452694 | $5.85\times10^{-5}$ | $1.68\times10^{-3}$ |
| 1 | rs4575098 | A | G | 0.228026 | 6.35743578 | $2.05\times10^{-10}$ | 11730923_x_at | *ADAMTS4* | -3.4327928 | $6.35\times10^{-4}$ | $1.48\times10^{-2}$ |
| 1 | rs4575098 | A | G | 0.228026 | 6.35743578 | $2.05\times10^{-10}$ | 11718419_at | *FCER1G* | -3.1782706 | $1.55\times10^{-3}$ | $3.09\times10^{-2}$ |
| 1 | rs2093760 | A | G | 0.191962 | 8.82495729 | $1.10\times10^{-18}$ | 11760294_a_at | *CD55* | -3.2667773 | $1.14\times10^{-3}$ | $2.46\times10^{-2}$ |
| 2 | rs4663105 | C | A | 0.411349 | 13.9449582 | $3.38\times10^{-44}$ | 11746895_a_at | *BIN1* | 5.42234123 | $8.27\times10^{-8}$ | $3.30\times10^{-6}$ |
| 2 | rs4663105 | C | A | 0.411349 | 13.9449582 | $3.38\times10^{-44}$ | 11741810_x_at | *BIN1* | 5.67610177 | $2.07\times10^{-8}$ | $9.63\times10^{-7}$ |
| 2 | rs4663105 | C | A | 0.411349 | 13.9449582 | $3.38\times10^{-44}$ | 11757435_x_at | *BIN1* | 5.67600123 | $2.07\times10^{-8}$ | $9.63\times10^{-7}$ |
| 2 | rs4663105 | C | A | 0.411349 | 13.9449582 | $3.38\times10^{-44}$ | 11719631_s_at | *BIN1* | 6.08815733 | $1.94\times10^{-9}$ | $1.28\times10^{-7}$ |
| 3 | rs184384746 | T | C | 0.00110872 | 5.69454687 | $1.24\times10^{-8}$ | 11715428_x_at | *ARF4* | 3.36531377 | $8.09\times10^{-4}$ | $1.84\times10^{-2}$ |
| 6 | rs6931277 | T | A | 0.156437 | -6.4930489 | $8.41\times10^{-11}$ | 11740691_x_at | *AIF1* | 3.81204871 | $1.51\times10^{-4}$ | $3.92\times10^{-3}$ |
| 6 | rs6931277 | T | A | 0.156437 | -6.4930489 | $8.41\times10^{-11}$ | 11731019_x_at | *AIF1* | 3.32915398 | $9.20\times10^{-4}$ | $2.05\times10^{-2}$ |
| 6 | rs6931277 | T | A | 0.156437 | -6.4930489 | $8.41\times10^{-11}$ | 11745266_x_at | *TAP2* | -6.3677094 | $3.60\times10^{-10}$ | $2.88\times10^{-8}$ |
| 6 | rs6931277 | T | A | 0.156437 | -6.4930489 | $8.41\times10^{-11}$ | 11741632_a_at | *TAP2* | -5.9088386 | $5.53\times10^{-9}$ | $2.94\times10^{-7}$ |
| 6 | rs6931277 | T | A | 0.156437 | -6.4930489 | $8.41\times10^{-11}$ | 11723059_a_at | *TAP2* | -3.1609819 | $1.64\times10^{-3}$ | $3.17\times10^{-2}$ |
| 6 | rs6931277 | T | A | 0.156437 | -6.4930489 | $8.41\times10^{-11}$ | 11723058_a_at | *TAP2* | -6.5032296 | $1.56\times10^{-10}$ | $1.34\times10^{-8}$ |
| 6 | rs6931277 | T | A | 0.156437 | -6.4930489 | $8.41\times10^{-11}$ | 11759642_x_at | *HLA-DRB1* | -3.9407455 | $8.99\times10^{-5}$ | $2.51\times10^{-3}$ |
| 7 | rs1859788 | A | G | 0.32433 | -7.9282106 | $2.22\times10^{-15}$ | 11755835_s_at | *GATS* | 3.18361271 | $1.52\times10^{-3}$ | $3.09\times10^{-2}$ |
| 7 | rs1859788 | A | G | 0.32433 | -7.9282106 | $2.22\times10^{-15}$ | 11722909_a_at | *GATS* | 10.3419453 | $2.50\times10^{-23}$ | $4.66\times10^{-21}$ |
| 7 | rs1859788 | A | G | 0.32433 | -7.9282106 | $2.22\times10^{-15}$ | 11756508_a_at | *TAF6* | -3.2251348 | $1.32\times10^{-3}$ | $2.79\times10^{-2}$ |
| 7 | rs1859788 | A | G | 0.32433 | -7.9282106 | $2.22\times10^{-15}$ | 11749146_x_at | *TAF6* | -3.7481712 | $1.94\times10^{-4}$ | $4.92\times10^{-3}$ |
| 7 | rs1859788 | A | G | 0.32433 | -7.9282106 | $2.22\times10^{-15}$ | 11728725_x_at | *TAF6* | -3.0149441 | $2.67\times10^{-3}$ | $4.97\times10^{-2}$ |
| 7 | rs1859788 | A | G | 0.32433 | -7.9282106 | $2.22\times10^{-15}$ | 11721727_x_at | *TAF6* | -3.8152085 | $1.49\times10^{-4}$ | $3.92\times10^{-3}$ |
| 7 | rs1859788 | A | G | 0.32433 | -7.9282106 | $2.22\times10^{-15}$ | 11730247_a_at | *PVRIG* | 4.6251114 | $4.51\times10^{-6}$ | $1.40\times10^{-4}$ |
| 7 | rs1859788 | A | G | 0.32433 | -7.9282106 | $2.22\times10^{-15}$ | 11736389_x_at | *TRIM4* | -5.9689044 | $3.91\times10^{-9}$ | $2.28\times10^{-7}$ |
| 7 | rs1859788 | A | G | 0.32433 | -7.9282106 | $2.22\times10^{-15}$ | 11736388_a_at | *TRIM4* | -5.9612934 | $4.08\times10^{-9}$ | $2.28\times10^{-7}$ |

| | | | | | | | | | | | |
|---|---|---|---|---|---|---|---|---|---|---|---|
| 7 | rs1859788 | A | G | 0.32433 | -7.9282106 | $2.22\times10^{-15}$ | 11732427_a_at | *ZCWPW1* | 3.16076728 | $1.65\times10^{-3}$ | $3.17\times10^{-2}$ |
| 7 | rs1859788 | A | G | 0.32433 | -7.9282106 | $2.22\times10^{-15}$ | 11760666_x_at | *ZKSCAN1* | 4.86984486 | $1.40\times10^{-6}$ | $4.89\times10^{-5}$ |
| 7 | rs1859788 | A | G | 0.32433 | -7.9282106 | $2.22\times10^{-15}$ | 11760665_at | *ZKSCAN1* | 5.46541079 | 6.56E-08 | $2.72\times10^{-6}$ |
| 7 | rs1859788 | A | G | 0.32433 | -7.9282106 | $2.22\times10^{-15}$ | 11732213_x_at | *ZKSCAN1* | -3.3226381 | $9.41\times10^{-4}$ | $2.06\times10^{-2}$ |
| 7 | rs7810606 | T | C | 0.485387 | -6.6200683 | $3.59\times10^{-11}$ | 11723373_a_at | *EPHA1* | 4.7393005 | $2.63\times10^{-6}$ | $8.90\times10^{-5}$ |
| 11 | rs2081545 | A | C | 0.382122 | -7.9728583 | $1.55\times10^{-15}$ | 11760710_a_at | *MS4A6A* | -4.7160648 | $2.94\times10^{-6}$ | $9.65\times10^{-5}$ |
| 11 | rs2081545 | A | C | 0.382122 | -7.9728583 | $1.55\times10^{-15}$ | 11749293_x_at | *MS4A6A* | -10.840538 | $2.64\times10^{-25}$ | $5.91\times10^{-23}$ |
| 11 | rs2081545 | A | C | 0.382122 | -7.9728583 | $1.55\times10^{-15}$ | 11716846_a_at | *MS4A6A* | -11.296622 | $3.63\times10^{-27}$ | $1.35\times10^{-24}$ |
| 11 | rs2081545 | A | C | 0.382122 | -7.9728583 | $1.55\times10^{-15}$ | 11732866_x_at | *MS4A4A* | -5.8223336 | $9.08\times10^{-9}$ | $4.61\times10^{-7}$ |
| 11 | rs2081545 | A | C | 0.382122 | -7.9728583 | $1.55\times10^{-15}$ | 11732865_a_at | *MS4A4A* | -5.5375868 | $4.44\times10^{-8}$ | $1.98\times10^{-6}$ |
| 11 | rs2081545 | A | C | 0.382122 | -7.9728583 | $1.55\times10^{-15}$ | 11751570_a_at | *MS4A4A* | -6.0298488 | $2.74\times10^{-9}$ | $1.70\times10^{-7}$ |
| 14 | rs12590654 | A | G | 0.33648 | -6.3912394 | $1.65\times10^{-10}$ | 11735593_a_at | *SLC24A4* | 5.52351067 | $4.79\times10^{-8}$ | $2.06\times10^{-6}$ |
| 14 | rs12590654 | A | G | 0.33648 | -6.3912394 | $1.65\times10^{-10}$ | 11735594_a_at | *SLC24A4* | 4.61336689 | $4.76\times10^{-6}$ | $1.44\times10^{-4}$ |
| 15 | rs442495 | C | T | 0.354342 | -6.065891 | $1.31\times10^{-9}$ | 11730885_a_at | *FAM63B* | 5.30470232 | $1.54\times10^{-7}$ | $5.75\times10^{-6}$ |
| 15 | rs442495 | C | T | 0.354342 | -6.065891 | $1.31\times10^{-9}$ | 11755773_x_at | *FAM63B* | 5.37386384 | $1.07\times10^{-7}$ | $4.13\times10^{-6}$ |
| 15 | rs117618017 | T | C | 0.124823 | 5.52218674 | $3.35\times10^{-8}$ | 11740603_a_at | *APH1B* | 6.87148987 | $1.48\times10^{-11}$ | $1.38\times10^{-9}$ |
| 15 | rs117618017 | T | C | 0.124823 | 5.52218674 | $3.35\times10^{-8}$ | 11740601_a_at | *APH1B* | 6.25273694 | $7.26\times10^{-10}$ | $5.41\times10^{-8}$ |
| 15 | rs117618017 | T | C | 0.124823 | 5.52218674 | $3.35\times10^{-8}$ | 11720067_a_at | *APH1B* | 6.10180361 | $1.79\times10^{-9}$ | $1.25\times10^{-7}$ |
| 15 | rs117618017 | T | C | 0.124823 | 5.52218674 | $3.35\times10^{-8}$ | 11720068_a_at | *APH1B* | 7.21238813 | $1.52\times10^{-12}$ | $1.54\times10^{-10}$ |
| 15 | rs117618017 | T | C | 0.124823 | 5.52218674 | $3.35\times10^{-8}$ | 11740602_s_at | *APH1B* | 7.43713411 | $3.23\times10^{-13}$ | $4.00\times10^{-11}$ |
| 16 | rs59735493 | A | G | 0.298476 | -5.4919099 | $3.98\times10^{-8}$ | 11723657_a_at | *ZNF668* | -3.649139 | $2.84\times10^{-4}$ | $6.90\times10^{-3}$ |
| 17 | rs113260531 | A | G | 0.126086 | 6.12333064 | $9.16\times10^{-10}$ | 11748594_a_at | *RABEP1* | -8.9138946 | $4.82\times10^{-18}$ | $7.70\times10^{-16}$ |
| 17 | rs113260531 | A | G | 0.126086 | 6.12333064 | $9.16\times10^{-10}$ | 11718616_s_at | *RABEP1* | 8.84651459 | 8.31E-18 | $1.16\times10^{-15}$ |

| | | | | | | | | | | | |
|---|---|---|---|---|---|---|---|---|---|---|---|
| 17 | rs113260531 | A | G | 0.126086 | 6.12333064 | $9.16\times10^{-10}$ | 11735601_at | *CHRNE* | 3.83501354 | $1.38\times10^{-4}$ | $3.75\times10^{-3}$ |
| 17 | rs113260531 | A | G | 0.126086 | 6.12333064 | $9.16\times10^{-10}$ | 11722495_a_at | *MYBBP1A* | -3.0364818 | $2.49\times10^{-3}$ | $4.71\times10^{-2}$ |
| 17 | rs113260531 | A | G | 0.126086 | 6.12333064 | $9.16\times10^{-10}$ | 11722554_at | *NUP88* | -3.5512468 | $4.11\times10^{-4}$ | $9.76\times10^{-3}$ |
| 17 | rs113260531 | A | G | 0.126086 | 6.12333064 | $9.16\times10^{-10}$ | 11747065_x_at | *NUP88* | -4.1539448 | $3.70\times10^{-5}$ | $1.09\times10^{-3}$ |
| 17 | rs113260531 | A | G | 0.126086 | 6.12333064 | $9.16\times10^{-10}$ | 11732435_at | *SCIMP* | 3.73900931 | $2.01\times10^{-4}$ | $4.99\times10^{-3}$ |
| 19 | rs111278892 | G | C | 0.147136 | 6.50193146 | $7.93\times10^{-11}$ | 11746548_s_at | *CNN2* | -7.2762348 | $9.85\times10^{-13}$ | $1.10\times10^{-10}$ |
| 19 | rs111278892 | G | C | 0.147136 | 6.50193146 | $7.93\times10^{-11}$ | 11723416_x_at | *CNN2* | 11.1991128 | $9.17\times10^{-27}$ | $2.56\times10^{-24}$ |
| 19 | rs111278892 | G | C | 0.147136 | 6.50193146 | $7.93\times10^{-11}$ | 11723414_a_at | *CNN2* | 12.0514456 | $2.33\times10^{-30}$ | $1.30\times10^{-27}$ |
| 19 | rs111278892 | G | C | 0.147136 | 6.50193146 | $7.93\times10^{-11}$ | 11723415_s_at | *CNN2* | 13.2170534 | $1.56\times10^{-35}$ | $1.74\times10^{-32}$ |
| 20 | rs6014724 | G | A | 0.0950416 | -6.1762414 | $6.56\times10^{-10}$ | 11739100_a_at | *CSTF1* | -4.6308011 | $4.39\times10^{-6}$ | $1.40\times10^{-4}$ |
| 20 | rs6014724 | G | A | 0.0950416 | -6.1762414 | $6.56\times10^{-10}$ | 11741029_x_at | *CSTF1* | -5.2978528 | $1.60\times10^{-7}$ | $5.77\times10^{-6}$ |
| 20 | rs6014724 | G | A | 0.0950416 | -6.1762414 | $6.56\times10^{-10}$ | 11718943_a_at | *AURKA* | -3.1969674 | $1.46\times10^{-3}$ | $3.01\times10^{-2}$ |

Abbreviations: A1: minor allele; A2: major allele; ADNI: Alzheimer's Disease Neuroimaging Initiative; Chr: chromosome; eQTL: expression quantitative trait loci; FDR: false discovery rate; GWAS: genome-wide association studies; MAF: minor allele frequency; $P_{eQTL}$: *P*-value of eQTL analysis; $P_{GWAS}$: *P*-value of GWAS; SNP: single nucleotide polymorphism; $T_{eQTL}$: *T*-value of eQTL analysis; Transcript ID: transcript identifier of Affymetrix Human Genome U219 Array; $Z_{GWAS}$: *Z*-value of GWAS

**Supplementary Table 2. Blood eQTL data generated from GWAS genotyping and blood-based RNA expression data in AddNeuroMed**

| Chr | SNP | A1 | A2 | MAF | $Z_{GWAS}$ | $P_{GWAS}$ | Transcript ID | Gene | $T_{eQTL}$ | $P_{eQTL}$ | FDR-corrected $P_{eQTL}$ |
|---|---|---|---|---|---|---|---|---|---|---|---|
| 1 | rs4575098 | A | G | 0.228026 | 6.35743578 | $2.05\times10^{-10}$ | ILMN_2123743 | *FCER1G* | -5.51947 | $5.64\times10^{-8}$ | $5.13\times10^{-6}$ |
| 1 | rs4575098 | A | G | 0.228026 | 6.35743578 | $2.05\times10^{-10}$ | ILMN_1658383 | *HSPA6* | -3.0937678 | $2.09\times10^{-3}$ | $3.57\times10^{-2}$ |
| 2 | rs4663105 | C | A | 0.411349 | 13.9449582 | $3.38\times10^{-44}$ | ILMN_1674160 | *BIN1* | 4.3979003 | $1.35\times10^{-5}$ | $5.22\times10^{-4}$ |
| 2 | rs4663105 | C | A | 0.411349 | 13.9449582 | $3.38\times10^{-44}$ | ILMN_2309245 | *BIN1* | 3.316668 | $9.82\times10^{-4}$ | $2.06\times10^{-2}$ |
| 6 | rs6931277 | T | A | 0.156437 | -6.4930489 | $8.41\times10^{-11}$ | ILMN_1808405 | *HLA-DQA1* | 8.42798982 | $4.35\times10^{-16}$ | $1.19\times10^{-13}$ |
| 6 | rs6931277 | T | A | 0.156437 | -6.4930489 | $8.41\times10^{-11}$ | ILMN_2157441 | *HLA-DRA* | -4.3701113 | $1.53\times10^{-5}$ | $5.22\times10^{-4}$ |
| 6 | rs6931277 | T | A | 0.156437 | -6.4930489 | $8.41\times10^{-11}$ | ILMN_1774074 | *RXRB* | 3.10664257 | $2.01\times10^{-3}$ | $3.57\times10^{-2}$ |
| 7 | rs1859788 | A | G | 0.32433 | -7.9282106 | $2.22\times10^{-15}$ | ILMN_1699631 | *GATS* | 7.86935366 | $2.49\times10^{-14}$ | 3.39E-12 |
| 7 | rs1859788 | A | G | 0.32433 | -7.9282106 | $2.22\times10^{-15}$ | ILMN_1768754 | *PILRB* | -4.4400456 | $1.12\times10^{-5}$ | $5.11\times10^{-4}$ |
| 7 | rs1859788 | A | G | 0.32433 | -7.9282106 | $2.22\times10^{-15}$ | ILMN_2323385 | *TRIM4* | 3.19269627 | $1.50\times10^{-3}$ | $2.93\times10^{-2}$ |
| 11 | rs2081545 | A | C | 0.382122 | -7.9728583 | $1.55\times10^{-15}$ | ILMN_1721035 | *MS4A6A* | -4.7802007 | $2.35\times10^{-6}$ | $1.28\times10^{-4}$ |
| 11 | rs2081545 | A | C | 0.382122 | -7.9728583 | $1.55\times10^{-15}$ | ILMN_2359800 | *MS4A6A* | -3.9721891 | $8.24\times10^{-5}$ | $2.25\times10^{-3}$ |
| 15 | rs117618017 | T | C | 0.124823 | 5.52218674 | $3.35\times10^{-8}$ | ILMN_1767816 | *APH1B* | 4.1199093 | $4.48\times10^{-5}$ | $1.36\times10^{-3}$ |
| 16 | rs59735493 | A | G | 0.298476 | -5.4919099 | $3.98\times10^{-8}$ | ILMN_2254635 | *ITGAX* | -3.519294 | $4.75\times10^{-4}$ | $1.18\times10^{-2}$ |
| 17 | rs113260531 | A | G | 0.126086 | 6.12333064 | $9.16\times10^{-10}$ | ILMN_1719622 | *RABEP1* | 5.27089797 | $2.07\times10^{-7}$ | $1.42\times10^{-5}$ |
| 19 | rs76320948 | T | C | 0.0307669 | 5.46447952 | $4.64\times10^{-8}$ | ILMN_2369785 | *SNRPD2* | -3.3724035 | $8.07\times10^{-4}$ | $1.84\times10^{-2}$ |
| 19 | rs76320948 | T | C | 0.0307669 | 5.46447952 | $4.64\times10^{-8}$ | ILMN_1665831 | *CLPTM1* | 2.98652997 | $2.97\times10^{-3}$ | $4.77\times10^{-2}$ |

Abbreviations: A1: minor allele; A2: major allele; Chr: chromosome; eQTL: expression quantitative trait loci; FDR: false discovery rate; GWAS: genome-wide association studies; MAF: minor allele frequency; $P_{eQTL}$: *P*-value of eQTL analysis; $P_{GWAS}$: *P*-value of GWAS; SNP: single nucleotide polymorphism; $T_{eQTL}$: *T*-value of eQTL analysis; Transcript ID: transcript identifier of Illumina Human HT-12 v3 Expression BeadChips; $Z_{GWAS}$: *Z*-value of GWAS

**Supplementary Table 3. Results of differential gene expression analysis between AD, MCI and CN in ADNI**

| Transcript ID | Gene | $T_{MCI}$ | $P_{MCI}$ | FDR-corrected $P_{MCI}$ | $T_{AD}$ | $P_{AD}$ | FDR-corrected $P_{AD}$ |
|---|---|---|---|---|---|---|---|
| 11720068_a_at | *APH1B* | 2.37950761 | 0.01762073 | 0.16739694 | 3.60610847 | 0.00033443 | 0.00635408 |
| 11740603_a_at | *APH1B* | 2.10584783 | 0.03559698 | 0.22544756 | 3.16482438 | 0.00162355 | 0.01542369 |
| 11716846_a_at | *MS4A6A* | 1.1665279 | 0.24382497 | 0.71737066 | 2.30155332 | 0.02167287 | 0.1167574 |
| 11741810_x_at | *BIN1* | -0.5361022 | 0.59206965 | 0.71737066 | -2.2336531 | 0.02584223 | 0.1167574 |
| 11719631_s_at | *BIN1* | -1.4925036 | 0.13604792 | 0.51698209 | -2.1331814 | 0.03328033 | 0.1167574 |
| 11757435_x_at | *BIN1* | -1.7392231 | 0.0824646 | 0.39170684 | -2.0914613 | 0.03687076 | 0.1167574 |
| 11746895_a_at | *BIN1* | -0.5659524 | 0.5716197 | 0.71737066 | -1.8577023 | 0.06365917 | 0.17278917 |
| 11755835_s_at | *GATS* | 3.08088286 | 0.00215024 | 0.04085449 | 1.64800038 | 0.09983143 | 0.23709964 |
| 11749293_x_at | *MS4A6A* | 0.93342924 | 0.35094182 | 0.71737066 | 1.46129947 | 0.14441209 | 0.30486997 |
| 11722909_a_at | *GATS* | 0.80371346 | 0.42185361 | 0.71737066 | 1.37139168 | 0.17072179 | 0.3243714 |
| 11748594_a_at | *RABEP1* | -1.0805145 | 0.28031023 | 0.71737066 | -1.2710846 | 0.20414909 | 0.35262115 |
| 11760710_a_at | *MS4A6A* | 0.22731267 | 0.82025144 | 0.86582097 | 1.1901364 | 0.23442331 | 0.37117024 |
| 11718419_at | *FCER1G* | -0.0111897 | 0.99107547 | 0.99107547 | 1.06608133 | 0.28677908 | 0.41913866 |
| 11720067_a_at | *APH1B* | -0.3513944 | 0.72540528 | 0.81074708 | 0.47572597 | 0.63442799 | 0.80384508 |
| 11718616_s_at | *RABEP1* | -0.6529023 | 0.51404833 | 0.71737066 | -0.4754644 | 0.63461453 | 0.80384508 |
| 11736388_a_at | *TRIM4* | -0.5187621 | 0.60410161 | 0.71737066 | 0.2555444 | 0.79838276 | 0.94807953 |
| 11740601_a_at | *APH1B* | -0.7431566 | 0.45765291 | 0.71737066 | 0.10536262 | 0.91612029 | 0.95095326 |
| 11736389_x_at | *TRIM4* | -0.8272241 | 0.40841083 | 0.71737066 | 0.06291794 | 0.94985102 | 0.95095326 |
| 11740602_s_at | *APH1B* | -0.8946098 | 0.3713238 | 0.71737066 | 0.06153327 | 0.95095326 | 0.95095326 |

*T* value and *P* values were obtained from analysis of covariance for diagnosis group (CN, MCI and AD) with adjustment of age and sex.
Abbreviations: AD: Alzheimer's disease; ADNI: Alzheimer's Disease Neuroimaging Initiative; CN: cognitively normal older adults; FDR: false discovery rate; MCI: mild cognitive impairment; $P_{AD}$: *P*-value for AD diagnosis; $P_{MCI}$: *P*-value for MCI diagnosis; $T_{AD}$: *T*-value for AD diagnosis; $T_{MCI}$: *T*-value for MCI diagnosis; Transcript ID: transcript identifier of Affymetrix Human Genome U219 Array
