# Supplementary material for: Dysregulated expression levels of APH1B in peripheral blood are associated with brain atrophy and amyloid-β deposition in Alzheimer’s disease
Source: Alzheimers Res Ther. 2021 Nov 3;13:183. doi: 10.1186/s13195-021-00919-z (PMC8567578; doi:10.1186/s13195-021-00919-z)

Supplementary Figure 1. GWAS quality control procedures for samples in the Alzheimer's Disease Neuroimaging Initiative cohort

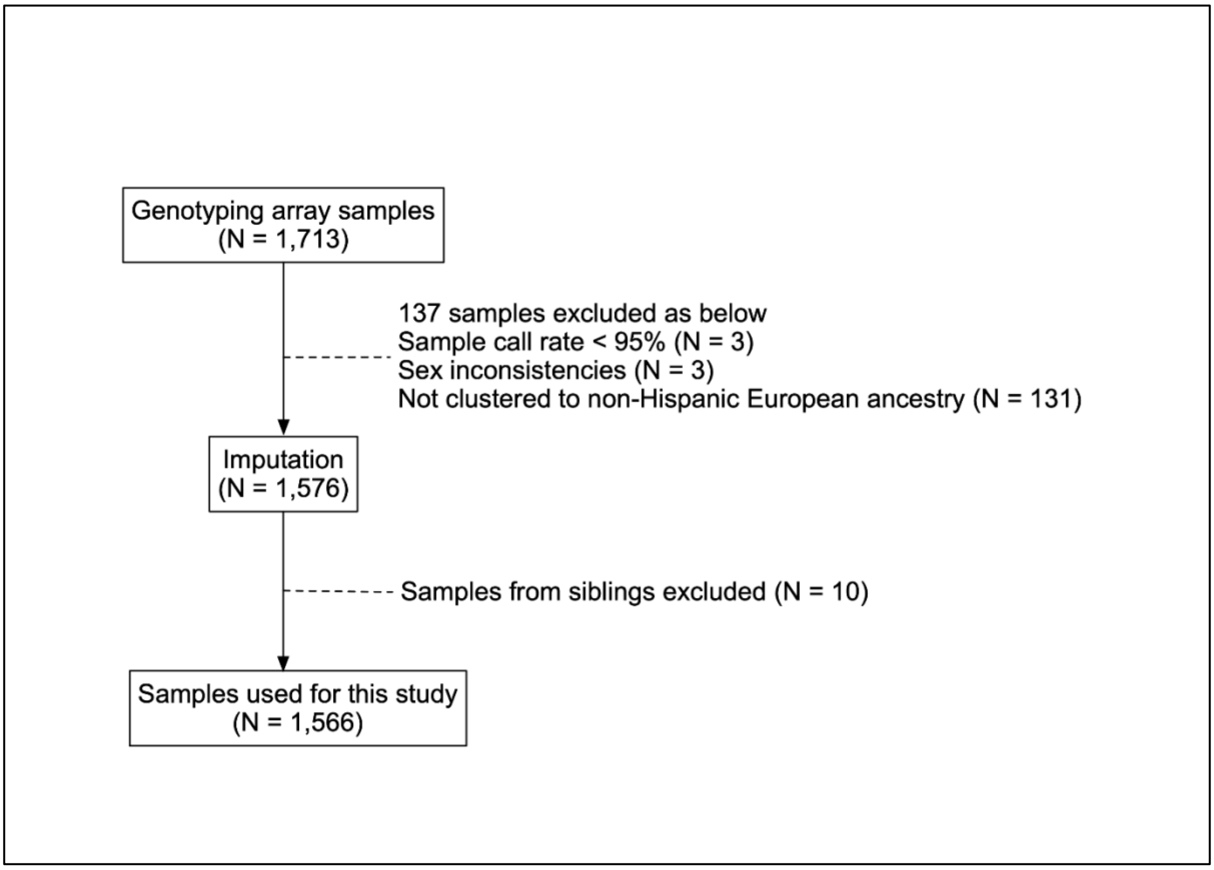

Abbreviation: GWAS: genome-wide association study.

Supplementary Figure 2. Quality control procedures of mRNA expression data for samples in the Alzheimer's Disease Neuroimaging Initiative cohort

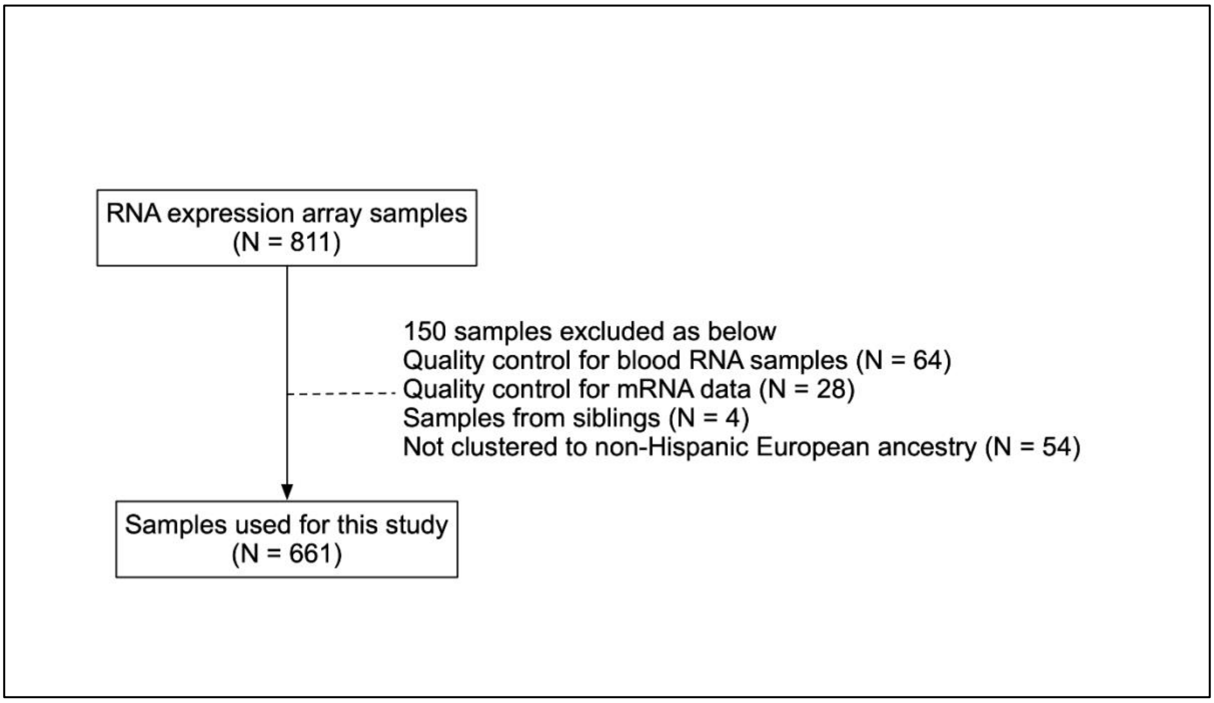

Supplement: Supplementary file 2 — Additional file 2: Supplementary Figure S1. GWAS quality control procedures for samples in the Alzheimer’s Disease Neuroimaging Initiative cohort. Supplementary Figure S2. Quality control procedures of mRNA expression data for samples in the Alzheimer’s Disease Neuroimaging Initiative cohort [file 13195_2021_919_MOESM2_ESM.pdf]
